# Effects of pre-extraction intermittent PTH administration on extraction socket healing in bisphosphonate administered ovariectomized rats

**DOI:** 10.1038/s41598-020-79787-w

**Published:** 2021-01-08

**Authors:** Jae-Young Kim, Hyo-Won Jang, Jung-In Kim, In-Ho Cha

**Affiliations:** 1 Department of Oral and Maxillofacial Surgery, Gangnam Severance Hospital, Yonsei University College of Dentistry, Seoul, South Korea; 2 Department of Oral and Maxillofacial Surgery, Sahmyook Dental Hospital, Seoul, South Korea; 3 Jung-In Dental Clinic, Seoul, South Korea; 4 Department of Oral and Maxillofacial Surgery, Oral Cancer Institute, Yonsei University College of Dentistry, 50, Yonsei-ro, Seodaemun-gu, Seoul, 03722 South Korea

**Keywords:** Dental diseases, Oral diseases

## Abstract

The purpose of this study was to investigate the effect of administering intermittent parathyroid hormone (iPTH) before tooth extraction versus after tooth extraction on the risk of developing MRONJ in experimental animal model. Twenty-five ovariectomized rats received 6 weeks of bisphosphonate therapy. They were classified into 3 groups, based on the timing of the medication, as Control, Pre-PTH and Post-PTH groups. For Control group, normal saline was administered before and after tooth extraction. iPTH was administered during 4 weeks before tooth extraction for Pre-PTH group and after tooth extraction for Post-PTH group. The animals were euthanized 8 weeks after tooth extraction. Macroscopic, histological, micro-computed tomography (micro-CT), and histomorphometric examinations were conducted. The incidences of impaired healing were 11.11% both in Pre-PTH and Post-PTH groups, which was lower than the Control group (42.86%). Bone healing in the extraction socket, based on micro-CT and histomorphometry evaluations, was best in Post-PTH and worst in Control group. The Pre-PTH group showed moderate healing pattern. Despite of limitations in this study, the authors identified Pre-PTH group seems to have positive effect on extraction socket healing. With regard to timing, administering iPTH after tooth extraction was superior to applying it before tooth extraction.

## Introduction

Bisphosphonates (BPs) inhibit bone resorption through several mechanisms such as inhibiting osteoclast formation and decreasing the bone turnover rate and apoptosis of osteoclasts^1,2^. In this regard, BPs have a good effect in treating bone metabolic diseases such as osteoporosis or preventing bone metastasis of malignant tumors. However, complications such as medication-related osteonecrosis of the jaw (MRONJ) have been reported after invasive dental treatment^3^.

Despite the complete absence of data showing that discontinuing anti-resorptive medications affects the occurrence of MRONJ, discontinuation of BPs is recommended to decrease the risk of MRONJ. A 2014 position paper by the American Association of Oral and Maxillofacial Surgery suggested a 2-month cessation of BPs as a prudent approach^4,5^. In an animal study, Zandi et al.^6^ reported 3 months of drug cessation might decrease incidence decrease of MRONJ development. However, there is still no clear criteria exist for the period of BP discontinuation. In addition, discontinuing BPs does not completely prevent MRONJ.

Intermittent parathyroid hormone (iPTH) administration, which has an anabolic effect, is widely used to manage intractable MRONJ^7,8^. The administration of iPTH with bone morphogenic protein-2 was recently reported in the management of MRONJ^9^. However, the role of iPTH to prevent MRONJ is relatively unknown, compared to its role in treating MRONJ.

Several studies have compared the effect of administering iPTH timing regarding surgical intervention with or without previous bisphosphonate treatment; however, the findings of these studies remain controversial^10,11^. Keskinruzgar et al.^10^ reported the risk of MRONJ development may decrease when PTH is administered before or immediately after tooth extraction with a greater number of osteoclasts when PTH was administered before or immediately after tooth extraction. However, based on the findings of a study by Kuroshima et al.^11^, no significant differences existed when iPTH was administered for the 7 days before tooth extraction and ridge preservation, compared with sterile saline injection (i.e., the control group).

The purpose of this study was to compare the effect of iPTH on extraction socket healing according to administration timing (i.e. before tooth extraction versus after tooth extraction) in bisphosphonate administered, ovariectomized rat model.

## Results

Twenty-five rats underwent ovariectomy, followed by an 8-week waiting period. Then, zoledronic acid (200 μg/kg) was administered for 6 weeks. The bisphosphonate administered, ovariectomized rats were randomly divided into three groups: the Control group, Pre-PTH group, and Post-PTH group. 80 μg/kg of parathyroid hormone (PTH) was administered daily for 4 weeks before or after tooth extraction according to the experimental groups. Same dosage of normal saline was administered when PTH was not administered. The rats were euthanized after 8 weeks after tooth extraction. No rats were expired during experimental periods.

### Effects of iPTH on the development of osteonecrosis of the jaw

The incidence of osteonecrosis of the jaw (ONJ) is summarized in Table 1. In macroscopic analysis, three (42.86%) of seven Control group rats had ONJ. One (11.11%) of nine rats had ONJ in the Pre-PTH group and in the Post-PTH group, although this difference was not statistically significant (*p* = 0.260). ONJ was histologically diagnosed in four (57.14%) of seven Control group rats. The incidence of MRONJ healing was 33.33% and 11.11% in the Pre-PTH and Post-PTH groups, respectively. However, the difference was not statistically significant (*p* = 0.146).

**Table 1:** Incidence of medication-related osteonecrosis of the jaw (MRONJ) development.

Group	Clinical analysis			Histologic assessment		
	Impaired healing	Incidence (%)	*p*-value	Impaired healing	Incidence (%)	*p*-value
Control (n = 7)	3	42.86	0.260	4	57.14	0.180
Pre-PTH (n = 9)	1	11.11		3	33.33	
Post-PTH (n = 9)	1	11.11		1	11.11	

Administration of intermittent PTH seemed to have a positive effect on extraction socket healing both in Pre-PTH and Post-PTH groups.

### Effects of iPTH on the bone healing of extraction socket

**Figure 1:**
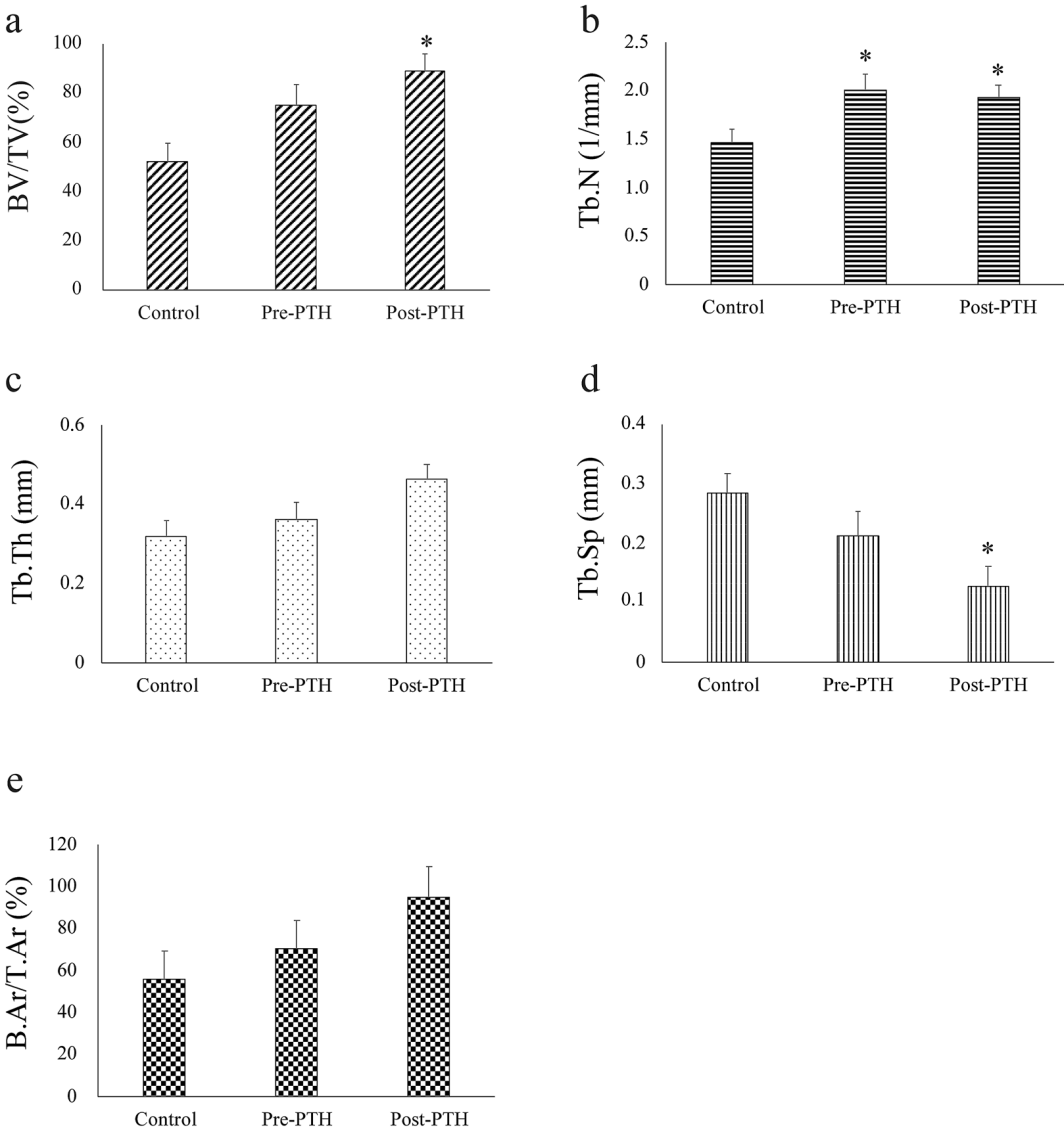
Effects on the extraction socket healing related to parathyroid hormone (PTH) administration. (**a**–**d**) Micro-computed tomography (micro-CT) analysis. In micro-CT analysis, the PTH-administered groups showed higher (**a**) bone volume/tissue volume (BV/TV), (**b**) trabecular number (Tb.N), and (**c**) trabecular thickness (Tb.Th), and (**d**) lower trabecular separation (Tb.Sp), compared with the Control group. (**e**) Histomorphometric analysis. The bone area/tissue area (B.Ar/T.Ar) shows a similar pattern as the BV/TV in the micro-CT analysis. *p < 0.05, versus the Control.

In the micro-CT evaluation, the BV/TV value was highest in the Post-PTH group (88.65%) and lowest in the Control group (52.26%) and was significantly different (*p* = 0.020). The value of BV/TV in the Pre-PTH group was 74.77% with close to the level of significance compare with Control group (*p* = 0.075) and with no significant difference when compared with Post-PTH group (*p* = 0.237). Tb.N value was higher in the Pre-PTH (2.01/mm) and Post-PTH (1.93/mm) groups than in the Control group (1.46/mm), and was also significantly different (*p* = 0.049). The Tb.Th value showed similar tendency with close to the level of significance (*p* = 0.060). The Tb.Sp value was lowest in the Post-PTH group and highest in the Control group with significant difference (*p* = 0.039) (Fig. 1a–d).

In histomorphometric analysis, B.Ar/T.Ar in the extraction socket was highest in the Post-PTH group (94.47%) and lowest in the Control group (55.76%) (Fig. 1e). However, the difference was not significant statistically (*p* = 0.198).

**Figure 2:**
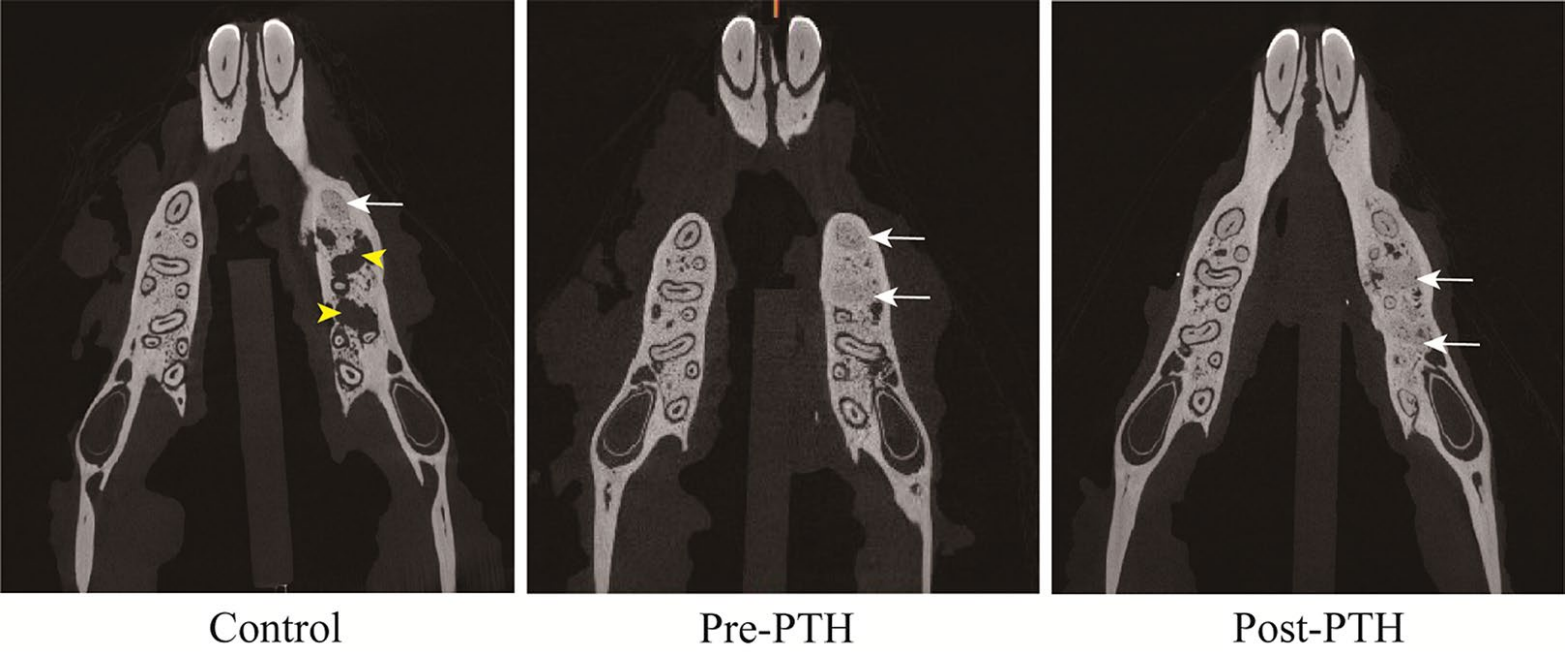
Representative images of micro-CT of each group. Insufficient bone healing was observed in control group (yellow arrowhead), whereas good bone healing of extraction socket was observed in pre-PTH and post-PTH groups (white arrow).

The micro-CT image of each group was presented in Fig. 2 and the actual data was summarized and presented in Table 2.

**Table 2:** Effects on the extraction socket healing related to parathyroid hormone (PTH) administration (The actual data of Fig. 1).

	Contol	Pre-PTH	Post-PTH	*p*-value
**Micro-CT analysis**				
BV/TV (%)	52.26 ± 7.16	74.77 ± 8.67	88.65 ± 6.96*	0.020
Tb.N (1/mm)	1.46 ± 0.15	2.01 ± 0.16*	1.93 ± 0.14*	0.049
Tb.Th (mm)	0.32 ± 0.04	0.36 ± 0.04	0.46 ± 0.04	0.060
Tb.Sp (mm)	0.29 ± 0.03	0.21 ± 0.04	0.13 ± 0.03*	0.039
**Histomorphometric analysis**				
B.Ar/T.Ar (%)	55.76 ± 13.44	70.49 ± 13.29	94.47 ± 14.90	0.198

*BV/TV* bone volume/tissue volume, *Tb.N* trabecular number, *Tb.Th* trabecular thickness, *Tb.Sp* trabecular separation, *B.Ar/T.Ar* bone area/tissue area.

*p < 0.05, versus the Control.

### Effects of iPTH on the trabecular bone of the proximal tibia

The effect of PTH administration on the proximal tibia can be found on Supplementary Fig. S1. Compared with the Control group, the Pre-PTH and Post-PTH groups had significantly higher values for BV/TV (*p* = 0.004) and Tb.Th (*p* = 0.048), and significantly lower values for Tb.Sp (*p* = 0.029). Compared with the Control group, the Pre-PTH group had significant higher values for Tb.N (*p* = 0.002). However, for all parameters, there were no significant differences between the Pre-PTH and Post-PTH groups (Supplementary Fig. S1b–e).

## Discussion

In 2001, Neer et al. reported the anabolic action of iPTH administration^12^. Since then, several investigators have reported the effectiveness of iPTH on the healing of fractures, after tooth extraction, periodontal healing, and in the treatment of MRONJ^7,13–16^. Intermittent parathyroid hormone administration could be considered as treatment modality for severe MRONJ. However, its effect on preventing MRONJ and extraction socket healing remains unclear^16–18^.

The purpose of this study was to compare the effect of iPTH on extraction socket healing according to administration timing (i.e. before tooth extraction versus its effect after tooth extraction) following bisphosphonate pre-treatment in experimental animal model. In this study, we considered that macroscopically impaired healing as MRONJ to have occurred or may occur in the future.

Park et al. reported rats had become osteopenic after ovariectomy compared with sham surgery^19^. Yoon et al. reported bone mass was significantly decreased 8 weeks after ovariectomy in rats^20^. Bisphosphonate is a drug for the treatment of osteoporosis. The rats underwent ovariectomy and BPs had been started after 8 weeks in order to reproduce this condition as much as possible.

Micro-CT analysis of the proximal tibia was conducted to observe the systemic effect of PTH administration. The Pre-PTH and Post-PTH groups had significantly higher values for BV/TV, Tb.N, and Tb.Th, and significantly lower value for Tb.Sp, compared with those of the Control group (*p* < 0.05). Thus, the exogenously administered PTH appears to have been functioning properly for bone metabolism in ovariectomized rats.

The incidence of ONJ after tooth extraction varies 60–95%, depending on the dose and duration of ZA injection^6,10,21–23^. In this study, 200 μg/kg of ZA was administered every week (1×/week), according to our previous study^24^. The dosage was decided considering following factors as Kim et al. reported: (1) the oncologically relevant zoledronate dosed in humans (67 μg/kg/4 weeks); (2) the relatively rapid bone metabolism of rodents; (3) the route of bisphosphonate administration; lower plasma concentration through intraperitoneal injection compared with intravenous injection; and (4) maximize exposure to bisphosphonate during relatively short experimental periods^24^. In the present study, the incidence of osteonecrosis of the jaw was 42.86%, 11.11% and 11.11% in the Control group, Pre-PTH group, and Post-PTH group, respectively. After the microscopic evaluation, MRONJ was diagnosed in 57.14%, 33.3%, and 11.1% of the Control group, Pre-PTH group, and Post-PTH group, respectively.

The dosage of PTH (80 ug/kg) was determined by reference to the study of Kuroshima et al.^25^ In their study, the reported 80 ug/kg of PTH therapy for 2 weeks after bisphosphonate combined with steroid and tooth extraction rescued necrotic lesions.

Micro-CT revealed that the degree of bony healing of the extraction socket was greater in the Post-PTH group than in the Pre-PTH group, and the degree of healing was significantly greater in these groups than in the Control group (*p* = 0.020). Histomorphometric analysis was conducted to verify the results of micro-CT and to confirm the correlation between microscopic analysis and micro-CT. The degree of bone healing observed on micro-CT images and with histomorphometric analysis showed a similar tendency, as shown in Fig. 1a,e. The degree of bone fill was greater in the extraction socket of the Pre-PTH group (94.47%) than in the Control group (55.76%); however, the difference was not significant in histomorphometric analysis (*p* = 0.198).

Although, histomorphometric analysis revealed no statistically significant difference, the healing pattern of the three groups observed with H&E staining showed different tendencies. In the Control group, favorable bone healing was observed in cases of “healing”. However, in cases of “MRONJ,” inflammatory cell infiltration with necrotic bone or severe surrounding bone destruction was observed. The Pre-PTH group showed varying results. The extraction socket healing occurred in mature bone with or without fibrotic tissue formation. The extraction socket was filled with inflammatory cells in “ONJ” cases. However, unlike in the Control group, there was little evidence of severe bone destruction in Pre-PTH group. Most rats in the Post-PTH group had excellent bone healing but without a substantial difference among the animals. Pre-PTH group and Post-PTH group also showed osteoclastic activity in extraction socket compared with Control group (Supplementary Fig. S3). We consider this phenomenon had an indirect effect of iPTH administration on bone healing^26^.

Several studies have compared the effect of administering iPTH before or after surgical intervention with or without previous bisphosphonate treatment; however, the findings of these studies remain controversial^10,11^. Keskinruzgar et al.^10^ reported a greater number of osteoclasts when PTH was administered before or immediately after tooth extraction, compared to when only ZA was administered. Keskinruzgar also reported the risk of osteonecrosis development may decrease when PTH is administered before or immediately after tooth extraction. However, based on the findings of a study by Kuroshima et al.^11^, no significant differences existed when iPTH was administered for the 7 days before tooth extraction and ridge preservation, compared with sterile saline injection (i.e., the control group). On the other hand, iPTH had an effect when administered during the 14-day period after the same procedure. Kuroshima reported that dynamic cellular responses such as inflammation after tooth extraction may contribute to the anabolic effect of iPTH^11^. The anabolic action of PTH is more powerful after surgical intervention than in undisturbed bone, and is enhanced where the bone metabolism rate is high^25^. We propose that the anabolic action of PTH increases because bone metabolism is activated after tooth extraction, compared with before tooth extraction.

This study was designed as pre-clinical animal experiment, it may be difficult to apply direct to clinical practice. It seems that Pre-PTH and Post-PTH group showed better healing tendency and positive effect on bone healing compared with control group. With regard to the timing of drug administration, iPTH administered after tooth extraction (i.e., Post-PTH group) had a better outcome, compared with iPTH administration before extraction (i.e., Pre-PTH group), whereas there was no significant difference when three groups were compared simultaneously.

In this study, the sample size was simply determined with reference to other experimental animal studies^10,15^. Maybe this sample size is not enough to generalize the effect of intermittent PTH administration before tooth extraction to prevent MRONJ development. In addition, some investigators have suggested that a low bone turnover rate induced by bisphosphonate can reduce the availability of osteoblasts, marrow-lining cells, and preosteoblasts, and the effects of continued bisphosphonate treatment reduce the PTH-induced bone formation^27,28^. Therefore, it is necessary to discontinue bisphosphonate for a certain period before initiating PTH treatment because PTH has a delayed effect when a person is pretreated with bisphosphonate^29^.

In the future, studies using a larger number of experimental animals or longer iPTH administration may be required.

## Materials and methods

### Animal model and study design (Fig. 3)

**Figure 3:**
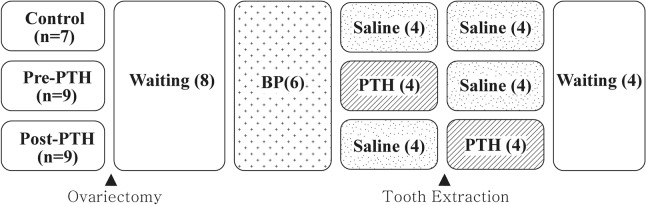
Experimental design. Rats (n = 25) underwent ovariectomy, followed by an 8-week waiting period. Zoledronic acid (200 μg/kg) was administered for 6 weeks. Before or after tooth extraction, 80 μg/kg of saline or parathyroid hormone (PTH) was administered daily, depending on the group. The parentheses refer to the period (weeks) taken for the experiments. *BP* bisphosphonate.

Twenty-five female Sprague–Dawley rats (Orientbio Co., Ltd. Seongnam, Korea) were used in this study. The mean weight was 232.9 g (range 201–262.5 g). They were randomly divided into three groups: the Control group, Pre-PTH group, and Post-PTH group. Animals were housed two rats per cage and were individually marked. The cages were placed in a room with filtered air at a temperature of 22 ± 2 °C and relative humidity of 50 ± 10%. A 12-h light/dark cycle was maintained. The animals were fed a normal rodent diet and water ad libitum. Animals were acclimated for 1 week before the beginning of the study. This study was approved by and performed in accordance with the guidelines of the Yonsei University Health System Institutional Animal Care and Use Committee (IACUC No. 2017–0063).

All rats were 11 weeks old and underwent ovariectomy at 12 weeks of age. An 8-week waiting period was used to induce an osteopenic condition, based on the procedure of Park et al.^19^ ant Yoon et al.^20^. Zoledronic acid (ZA; 200 μg/kg; Zometa Ready, Novartis, Switzerland) was injected into peritoneum for 6 weeks (1×/week, weekly), based on the findings in our previous study^24^. Then, the rats were divided into three groups as Control group, Pre-PTH group and Post-PTH group.

After 6 weeks of ZA administration, an analog of parathyroid hormone (PTH) (Bachem, Torrance, CA, USA), was subcutaneously administered daily at a dose of 80 µg/kg in the Pre-PTH group for 4 weeks^25^. During that period, same dose of normal saline was administered for Control and Post-PTH groups. After 4 weeks (at the end of PTH injection period in the Pre-PTH group), the left mandibular first (M1) and second molars (M2) were extracted, using the procedure reference with previous protocol^24^. The movable broken roots were scraped once or twice to remove by using a sharp explorer. Excessive forces in extraction socket was avoided as far as possible to prevent additional trauma. No other equipment such as a microdrill was used. An additional suture was not applied and the extraction socket was left open, as is practiced in human beings. Intramuscular injection of meloxicam (1.5 mg/kg body weight; Metacam, Boehringer Ingelheim, Germany) was administered for pain control at the time of tooth extraction.

After tooth extraction, PTH analogue (80 μg/kg/day) was administered for 4 weeks. For Control group and Pre-PTH group, same dose of normal saline was injected.

### Macroscopic evaluation of the extraction socket

All rats were euthanized 8 weeks after the tooth extraction. The mandible was dissected and fixed using 10% formalin solution. An extraction socket covered by normal mucosa without dehiscence was defined as having a normal healing state. Exposure of the underlying bone or mucosal dehiscence was defined as impaired healing, which considered as MRONJ to have occurred or may occur in the future. Mucosal hyperemia or granulation tissue formation on the extraction socket was also defined as impaired healing^22^.

### Microscopic computed tomography

Micro-computed tomography (micro-CT; Skyscan 1173, Konitch, Belgium) images were scanned at 13.85-μm pixels (for the mandible) and 15.98-μm pixels (for the tibia) with an energy level of 90 kV. The data were evaluated using CTAn software (Skyscan, Konitch, Belgium). To assess the systemic effect of the administered PTH, the center of trabecular bone between 1.2 and 3.5 mm from the growth plate of the proximal tibia was analyzed. The region of interest (ROI) was set as a circle of 2.0 mm × 2.0 mm at the 1.2 mm site and a circle of 1.5 mm × 1.5 mm circle at the 3.5 mm site (Supplementary Fig. S2a)^25^. To evaluate the extraction socket, the ROI was set on the center of the extracted site in the coronal view (Supplementary Fig. S2b). Seventy images of the M2 distal root (0.7 mm × 0.7 mm circle) and M1 distal root (1.0 × 1.0 mm circle), and 80 images of the M1 mesial root (0.8 mm × 0.8 mm circle) were analyzed with regard to the mesiodistal length of each root. Bone volume/tissue volume (BV/TV [%]), trabecular number (Tb.N [1/mm]), trabecular thickness (Tb.Th [mm]), trabecular separation (Tb.Sp [mm]), and bone marrow density were assessed in each group.

### Histology and histomorphometry

**Figure 4:**
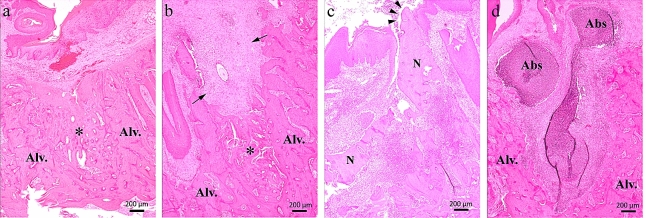
Representative images of (**a**,**b**) healing and (**c**,**d**) MRONJ, based on hematoxylin and eosin (H&E) staining (magnified × 50). (**a**,**b**) The extraction socket is filled with bone completely or partially (asterisk mark). Black arrow indicates fibrotic healing states. (**c**) The extraction socket shows impaired healing with ulcerative epithelium (arrowhead) and necrotic bone (N). (**d**) The extraction socket is filled with inflammatory cells, although surrounding bone is intact. The scale bar (black line) indicates 200 μm. *Alv.* alveolar bone, *N* necrotic bone, *Abs* abscess.

All specimens were fixed with 10% formalin solution for 2 weeks and decalcified in 10% ethylenediaminetetraacetic acid (pH 7.4) for 3 weeks at room temperature. Samples were embedded in paraffin and sectioned with 3-μm thickness. Samples were stained with hematoxylin and eosin (H&E). Imparied healing was diagnosed if any of the following criteria were met: (1) the presence of an ulcerative lesion with exposed and necrotic bone and/or osteolysis, (2) the presence of pseudoepitheliomatous-like hyperplasia of the epithelium accompanied by inflammatory cell infiltration, and (3) the presence of sequestrum and bacterial colonies^24^. An ulcerative lesion was defined when the epithelium on the extraction socket was not fully covered (Fig. 4a–d).

Images were captured using a microscope (Axio Imager 2; Zeiss, Göttingen, Germany) and analyzed with image analysis software (MetaMorph, ver 7.8; Molecular Devices, Sunnyvale, CA, USA). Histomorphometric evaluation was focused on the extraction socket areas. The tissue area (T.Ar [mm^2^]) and bone area (B.Ar [mm^2^]) were measured. The B.Ar/T.Ar ratio (%) was calculated to normalize the parameters.

### Statistical analysis

Statistical analysis was conducted using a statistical software program (SPSS 23.0; IBM, Armonk, NY). Mucosal healing at the macroscopic and microscopic levels was compared using Fisher’s exact test. In the micro-CT analysis of tibia, one-way ANOVA was used to compare the 3 groups when the distribution satisfied the normality assumption. Otherwise, the Kruskal–Wallis test was used to compare the groups. Post-hoc test with Bonferroni’s method was performed to compare groups in case of significant difference after ANOVA. In the micro-CT analysis and histomorphometric analysis of mandible, mixed model was used to compare the 3 groups. Pairwise comparison was performed with LSD method. Values of *p* < 0.05 indicated a significant difference and 0.05 ≤ *p* < 0.1 was considered a trend close to significance to increase the sensitivity to detect potential selection bias.

## Conclusion

Despite of limitations in this study, the authors identified iPTH administration before tooth extraction seems to have a positive effect on extraction socket healing. With regard to timing, administering iPTH after tooth extraction was superior to applying it before tooth extraction.

## Supplementary Information

Supplementary Figure S1.

Supplementary Figure S2.

Supplementary Figure S3.

Supplementary Legends.
