# Supplementary figures and images for: Effects of pre-extraction intermittent PTH administration on extraction socket healing in bisphosphonate administered ovariectomized rats
Source: Sci Rep. 2021 Jan 8;11:54. doi: 10.1038/s41598-020-79787-w (PMC7794385; doi:10.1038/s41598-020-79787-w)

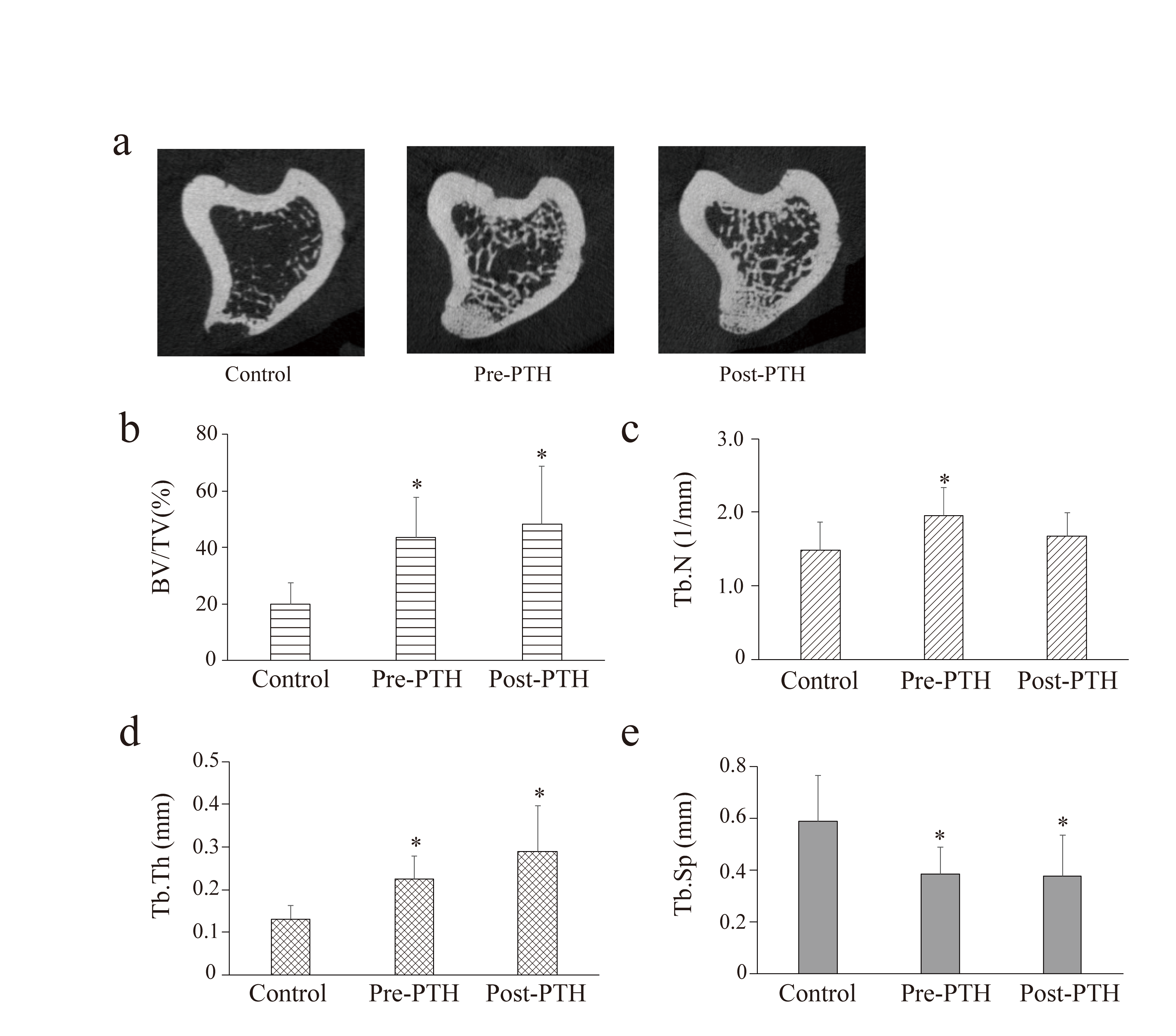

Supplement: Supplementary file 1 — Supplementary Figure S1. [file 41598_2020_79787_MOESM1_ESM.tif]

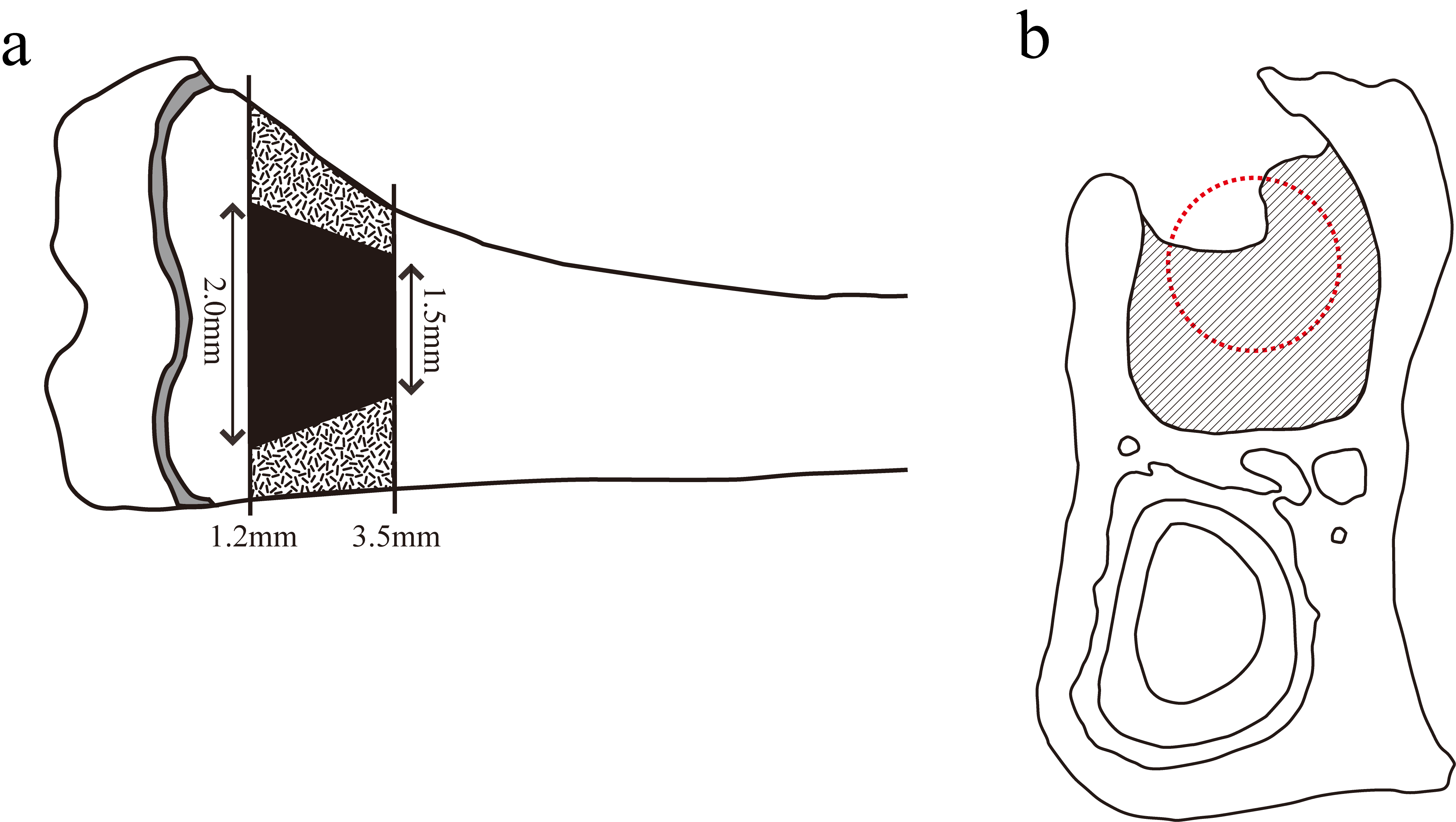

Supplement: Supplementary file 2 — Supplementary Figure S2. [file 41598_2020_79787_MOESM2_ESM.tif]

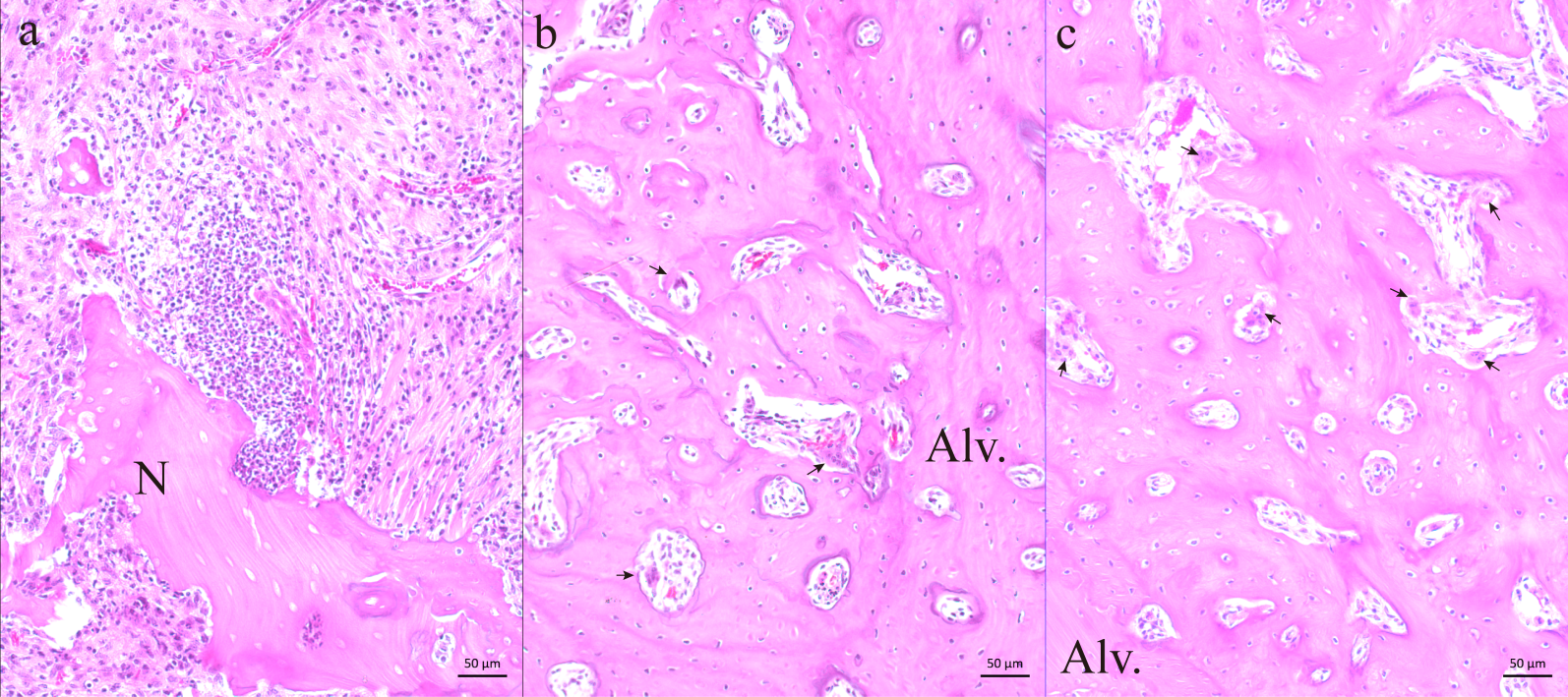

Supplement: Supplementary file 3 — Supplementary Figure S3. [file 41598_2020_79787_MOESM3_ESM.tif]
