# Supplementary material for: Effects of pre-extraction intermittent PTH administration on extraction socket healing in bisphosphonate administered ovariectomized rats
Source: Sci Rep. 2021 Jan 8;11:54. doi: 10.1038/s41598-020-79787-w (PMC7794385; doi:10.1038/s41598-020-79787-w)
Supplement: Supplementary file 4 — Supplementary Legends. [file 41598_2020_79787_MOESM4_ESM.pdf]

Effects of pre-extraction intermittent PTH administration on extraction socket healing in bisphosphonate administered ovariectomized rats

Jae-Young Kim[1], Hyo-Won Jang[2], Jung-In Kim[3], In-Ho Cha[4]

[1]Assistant professor, Department of Oral and Maxillofacial Surgery, Gangnam Severance Hospital, Yonsei University College of Dentistry, Seoul, Korea

[2]Clinical dentist, Department of Oral and Maxillofacial Surgery, Sahmyook dental hospital, Seoul, Korea

[3]Director, Jung-In Dental Clinic, Seoul, Korea

[4]Professor, Department of Oral and Maxillofacial Surgery, Oral Cancer Institute, Yonsei University College of Dentistry, Seoul, Korea

Corresponding author

In-Ho Cha

Department of Oral and Maxillofacial Surgery,

Yonsei University College of Dentistry

50, Yonsei-ro, Seodaemun-gu, Seoul, 03722, Korea

Tel: +82-2-2228-3140

Fax: +82-2-2227-8022

E-mail: cha8764@yuhs.ac

Supplementary Fig. 1 Effects on the proximal tibia related to parathyroid hormone (PTH) administration (**a**) Representative cross-sectional images of each group. The PTH-administered groups have a higher (**b**) bone volume/tissue volume (BV/TV) percentage, (**c**) trabecular number (Tb.N), and (**d**) trabecular thickness (Tb.Th), and (**e**) lower trabecular separation (Tb.Sp), compared with the Control group.

* $p < 0.05$, versus the Control

Supplementary Fig. 2 (**a**) Region of interest (ROI) in micro-computed tomography (micro-CT) analysis of the proximal tibia The ROI is set between 1.2 mm and 3.5 mm from the growth plate to identify the systemic effect of administering parathyroid hormone (PTH). (**b**) Micro-CT analysis of the socket. The ROI is focused on the

center of the extraction socket (red circle). The black oblique line indicates the extraction socket healing area.

Supplementary Fig. 3 Representative images of experimental groups based on H&E images (magnified x200). (a) Control group, **(b)** Pre-PTH group, **(c)** Post-PTH group. A number of inflammatory cells with surrounding necrotic bone was observed in Control group. Various number of osteoclast (arrow) were observed in Pre-PTH and Post-PTH groups in extraction socket. The scale bar (black line) indicates 50μm.

Alv., Alveolar bone; N, Necrotic bone
